# A framework and a measurement instrument for sustainability of work practices in long-term care

**DOI:** 10.1186/1472-6963-11-314

**Published:** 2011-11-16

**Authors:** Sarah S Slaghuis, Mathilde MH Strating, Roland A Bal, Anna P Nieboer

**Affiliations:** 1 Institute for Health Policy and Management, Erasmus University Rotterdam, P.O. BOX 1738, 3000 DR, Rotterdam, the Netherlands

## Abstract

**Background:**

In health care, many organizations are working on quality improvement and/or innovation of their care practices. Although the effectiveness of improvement processes has been studied extensively, little attention has been given to sustainability of the changed work practices after implementation. The objective of this study is to develop a theoretical framework and measurement instrument for sustainability. To this end sustainability is conceptualized with two dimensions: routinization and institutionalization.

**Methods:**

The exploratory methodological design consisted of three phases: a) framework development; b) instrument development; and c) field testing in former improvement teams in a quality improvement program for health care (N _teams _= 63, N _individual _= 112). Data were collected not until at least one year had passed after implementation.

Underlying constructs and their interrelations were explored using Structural Equation Modeling and Principal Component Analyses. Internal consistency was computed with Cronbach's alpha coefficient. A long and a short version of the instrument are proposed.

**Results:**

The χ^2^- difference test of the -2 Log Likelihood estimates demonstrated that the hierarchical two factor model with routinization and institutionalization as separate constructs showed a better fit than the one factor model (p < .01). Secondly, construct validity of the instrument was strong as indicated by the high factor loadings of the items. Finally, the internal consistency of the subscales was good.

**Conclusions:**

The theoretical framework offers a valuable starting point for the analysis of sustainability on the level of actual changed work practices. Even though the two dimensions routinization and institutionalization are related, they are clearly distinguishable and each has distinct value in the discussion of sustainability. Finally, the subscales conformed to psychometric properties defined in literature. The instrument can be used in the evaluation of improvement projects.

## Background

It is unclear how health care organizations can sustain changed work practices [1]. Although studies on quality improvement and organizational change have yielded important insights in improvement processes, they also seem to have a strong focus on effectiveness of projects and outcome indicators. As a result of this, evidence on effectiveness of actual work practices often has not been obtained [2]. Moreover, many studies analyze improvement processes within the boundaries of projects only (ibid), without noting effectiveness afterwards. In sum, insight into sustainability of work practices appears to be lacking. In this study, we developed a framework and measurement instrument for sustainability; after implementation.

The framework is centered on work practices, which can be defined as patterns of actions to perform multiple, often interrelated or even interdependent, tasks. The framework is founded on the idea that work practices can be described in terms of 'organizational routines' as theorized by Feldman and Pentland [3-5]. An organizational routine is defined as *'repetitive, recognizable pattern of interdependent actions, carried out by multiple actors' *(ibid). Like work practices, we can describe *changed *work practice also in terms of --changed or new-- organizational routines. This approach may be particularly interesting in the domain of health care, where work practices typically are dynamic and require improvisation as well as 'following the rules'. Sustainability can then be seen as a dynamic process in which actors in a targeted work practice develop and/or adapt the organizational routines to a new work method. This process can also be described as routinization: through the development of organizational routines a new work method becomes part of everyday activities [6,7]. This process also involves learning processes at different levels in the organization [8-10], as there is more to the daily performance of a work practice than just routinization. Organizational routines cannot be sustained without providing the conditions that support and enable the performance. This is institutionalization, understood as the gradual adaptation of the organizational context, including structures and processes, to the new work practice [6,7,11-13]. Although routinization and institutionalization are often taken to be almost synonymous, we propose that each concept has its distinct value in the discussion on sustainability. Where routinization covers the process in which the actions are shaped and steered, institutionalization extends to the embedding of a work practice in the organization, emphasizing the conditional aspects.

**Figure 1:**
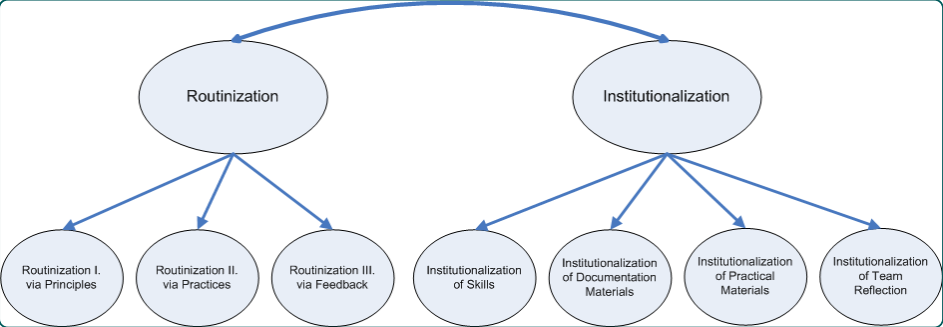
**Graphic representation of the framework**.

These two concepts are understudied in the domain of quality improvement and organizational change in health care. The purpose of this study is to further the conceptualization of sustainability with these concepts and to develop a measurement instrument, as can be seen in Figure 1. For each concept, several sub dimensions are defined, three for routinization and four for institutionalization (seven in total). We will elaborate on these first before presenting the methods.

### Routinization

Although the term routinization is sometimes used in studies on sustainability it is hardly ever defined or operationalized. We propose to take routine theory as starting point for such a conceptualization. According to Feldman and Pentland, organizational routines have a dual nature, which implies that *principles and the practices mutually form each other *[3]. On the one hand the organizational routine is constituted in the form of *a set of principles*; principles that the actors know and use to guide and explain their actions in the routine. On the other hand it is seen as it is performed *in practice: *through the performances, actors develop a shared 'formal' understanding (and language) as well as tacit knowledge of what needs to be done in a targeted situation [3-5,9]. Furthermore, actors can adjust the principles in light of their experiences and the insights gained through practice. It is obvious that reflection, monitoring and feedback during performance are very important in this process. For these reasons, Feldman and Pentland argue that organizational routines are "generative systems", with "internal structures and dynamics in which flexibility and adaption are equally important as stability" [3,4]. Consequential, organizational routines can also be a source of change.

We can now redefine routinization: this involves the sustenance of the organizational routine(s) for a work practice through the mutual reinforcement of principles and practices. In short, sustaining an organizational routine requires cultivating both principles and practices. The bidirectional relation might be useful to deepen our understanding of routinization as a dynamic, continuous process as in each performance actors align their actions to both the principle and the situation, while at the same they adjust the principles.

In this perspective, three sub dimensions for routinization can be deduced. The first sub dimension involves how principles form practices, i.e. the ways in which the principles are used to guide, account for and refer to the practices pertaining to the organizational routine. The second sub dimension regards how practices form principles, i.e. the ways in which the practices serve to create, maintain and modify the principles. Last, the third sub dimension concerns the collective monitoring and, in particular, the exchange of feedback on performance in practice. Routinization thus involves a mix of learning processes, including double and triple loop learning [14,15].

### Institutionalization

To reiterate, we define institutionalization as the gradual adaptation of the organizational context, including structures and processes, to the new work practice. In our framework we integrate the concept of institutionalization with the concept of routinization just presented [6,7,16]. This integration is achieved by directing the most elementary description of institutionalization at *the required supporting conditions for the targeted organizational routines*. We therefore focus on four sub dimensions which directly facilitate the performance of an organizational routine: institutionalization of skills, documentation materials, practical materials, and reflection. Although we recognize that Yin's conceptualization also contains elements such as rewarding systems, financial management of resources, HRM, planning and control cycles, etc., in our framework these are considered prerequisite to the four dimensions, rather than indicators of institutionalization in their own right. What follows is a description of the four sub dimensions.

First, new *skills *may be required to perform a new work practice. To sustain performance these should be provided, monitored, cultivated, and if necessary updated. On an institutional level this involves several organizational structures and processes: offering feedback on the skills, offering training, setting demands in job advertisements, monitoring via performance interviews, and so on. Next, organizational routines require many different materials for the actual performance, especially care practices. Two types of materials can be distinguished in form and function. *Practical materials *serve a primary function for the work practice. Some examples are practical tools or medical instruments, but also patient records. In contrast, *documentation materials *serve a more secondary function by offering extended memory on the organizational routine and supporting learning processes. Examples are protocols, manuals, care plans, etc. These usually contain formal explicit information on work practice related professional knowledge and skills. The last sub dimension, *team reflection*, refers to formal, purposive forms of reflection and monitoring of the quality of performance between professionals. Important for sustainability is a shared understanding of the main principles to monitor the actions during performance [4,17]. This understanding can be developed through institutionalized attention for the work practice in the form of 'shared reflection practices' outside performance.

Having introduced the framework we can move on to the development of the measurement instrument and the field testing. The analyses will have a twofold focus: 1. We will investigate the sub dimensions and the validity of items in the respective subscales. 2. We will explore the underlying constructs and their interrelations for the two dimensions routinization and institutionalization.

## Methods

### Sample and data collection

Field testing has been done with a follow-up study on the work methods developed in a quality improvement program in the Netherlands entitled Care for Better. The program was based on Breakthrough Methodology. Participating organizations were nursing homes, elderly homes, home care and care for disabled. In the years 2006-2008 seven projects were performed: pressure ulcers, eating and drinking, prevention of sexual abuse, client autonomy, medication safety, fall prevention, and prevention of (social) behavioral problems. In each project, improvement teams developed small practical interventions for care practices.

This follow-up study is part of a larger evaluation study on the Care for Better program [18]. At the end of April 2009 all former members of improvement teams who had finished the program more than a year ago were invited to participate in the follow-up study. Improvement teams usually consist of five members, a questionnaire for each member was offered. In the following weeks the researcher telephoned the contact persons to answer questions, map problems and encourage participation.

Of the 171 teams who received the questionnaires, 73 teams participated and 127 questionnaires were returned. The team response rate was 33% (73/171). This is comparable to the response rate in the data collection at the end of the projects [18,19]. We compute the individual response rate for an expected maximal return of approximately 2.5 respondents per team (50% of the formal maximum). On the individual level, the response rate is 30% (127/428). Fifteen cases from ten teams were excluded because of missing data. The data for analysis included 112 respondents from 63 teams. The average number of respondents per team was 2.2 at the end of the projects, and 1.6 in the current sample. Reasons for not participating in the study were mostly related to organizational dynamics in the field: high employee turnover and many team members now held other jobs. Others did not participate owing to lack of time, reorganization or other adverse organizational conditions.

### Instrument development

The conceptualization presented above pertains to a larger theoretical framework we are developing on sustainability and spread. This larger framework was based on a literature review on a range of themes, including: sustainability in health care organizations, as well as organizational change, spread, diffusion, and effectiveness of improvement processes. In addition, the framework has been discussed several times in our multidisciplinary evaluation research team.

For each sub dimension we designed a scale of 5-10 statements describing several practical aspects, to be evaluated on a five point Likert scale, ranging from '1: I don't agree at all' to '5: I agree very much', including the option 'I don't know'.

The content validity was assessed by the authors and 11 experts who all reviewed a draft version in relation to their specialism. The experts included 1) six members of our research team, 2) four other scholars in health policy and management, and 3) a former collaborative project leader.

The majority of the experts had actually worked in long-term care organizations, mostly as care professionals, such as nurse, occupational therapist, and a dietician, but also as quality staff or in a management position. About half of the experts also had practical professional experience in organizing quality improvement projects.

### Scales for routinization and institutionalization

#### Routinization

Three subscales were construed. The items are included in Additional file 1. Routinization I (10 items): Principle forming Practice, asks for the extent to which everybody knows how to perform the new work practice. Routinization II (8 items) asks if there are variations in practice and if the practices have led to new variations in the principles. Routinization III (5 items) represents the role of feedback on performance of the work practice and characterizes direct informal forms of such feedback.

#### Institutionalization

We construed a subscale for each of the proposed four sub dimensions of institutionalization; see Additional file 1. Institutionalization of Skills (8 items): this subscale centers on cultivating and evaluating required skills. Institutionalization of Documentation Materials (9 items); this subscale assesses availability and use of documentation materials for the work practice. Institutionalization of Practical Materials (7 items): this subscale assesses availability and use of materials such as medical instruments, diagnostic tests, as well as organizational instruments, such as work timetables or information systems for individual care plans.

Institutionalization of Team Reflection (5 items): this subscale focuses on the formalized evaluation practices amongst practitioners in teams.

### Statistical analyses

We present the main statistical results in two phases: 1) analyses of the initial set of items and the construction of a long version and 2) the construction of a short version.

The analyses were done in several steps. First, we studied the structure and content of the subscales and the individual items. Second, we assessed construct validity with structural equation modeling (SEM) and we explored to what extent the distinction between the two dimensions routinization and institutionalization is relevant (compared with a one-dimensional model). Aside from the analyses reported, several possible structures in the data were explored with principal component analyses (PCA). A selection of the PCA results is offered in Additional file 2. Third, the reliability of the subscales was assessed in terms of internal consistency with Cronbach's alpha. Finally, bivariate correlations were computed between the subscales and between the short and the long version of the instrument. We will now elaborate on the methodological decisions relevant for our analyses.

#### Data preparation

This study is based on individual level analyses of the data. We tested intra class coefficients to control for team level variance; for Routinization, ICC = .05, F(62,38) = 1.08; and for Institutionalization, ICC = .20, F(62,38) = 1.41 (both n.s.; variables were based on the long version). This means no evidence is found for a significant team level effect. Secondly, for the initial modeling with 52 items, list wise deletion of cases with missing data resulted in a small sample, N = 33. To be able to analyse the instrument integrally, i.e. with 52 items, we decided to impute missing data with the Expectation Maximization-algorithm provided in LISREL [20-22].

#### Model testing

All items were screened with descriptive statistics and missing values analysis. Then the structure of the measurement instrument was analyzed in a confirmatory factor analysis, see Figure 1. For the SEM a measurement model was construed, which comprised the estimation of factor loadings of the items on intended first order factors: Routinization I, Routinization II, Routinization III, Institutionalization of Skills, Institutionalization of Documentation, Institutionalization of Materials and Institutionalization of Team Reflection. No correlations between first order factors were allowed in this analysis. The hierarchical model in SEM, then, regards the relations between the seven factors and the two second order factors, Routinization and Institutionalization, see also Figure 1. With the factor loadings of the items and modification indices we verify the latent constructs indicated by the items to validate the subscales.

We compared the proposed hierarchical second order structure (2Fmodel) with one second order factor 'Sustainability' (1Fmodel) versus a model with no second order factors (0Fmodel).

#### SEM criteria

All structural equation models were computed with covariance-variance matrices and ML-estimation methods. In these analyses no correlations between measurement errors of items were allowed within or across subscales. Though the error variances are likely to correlate, we had no conceptual argument for interpreting relations outside the model-implied relations. For this reason we refrained from estimating any extra relation to enhance model fit. All model comparisons were based on χ^2^-difference tests of the -2Log Likelihood ratios at a significance level α = 0.05. For assessing goodness of fit, we reported commonly used indices [20,23,24]: the likelihood ratio χ^2^, Steiger - Lind's root mean square error of approximation (RMSEA) and its 90% confidence interval, comparative fit index (CFI), and the standardized root mean square residual (SRMR). The likelihood ratio χ^2 ^is considered a badness-of-fit index related to the probability that the model has perfect fit in the population; the lower the value, the better the fit. The RMSEA is a population based fit index derived from the likelihood ratio that is adjusted for parsimony. For a good model fit the RMSEA values should be low and are recommended to range between 0.08 and 0.05. In the CFI the differences between the independence model and estimated model are quantified and naturally these should be small. The CFI values should therefore range between 0.90 and 1.0. In addition, since some readers may be more acquainted with the Tucker-Lewis index (NNFI), the results for this index were included in Additional file 3; this index resembles the CFI and refers to the difference with the independence model while adjusting for parsimony. Lastly the SRMR indicates the goodness-of-fit in terms of covariance residuals, which should approximate zero. Good fit is indicated by SRMR values lower than 0.08.

#### Item selection

Items were selected using the following criteria: 1) factor loadings, modification indices, and reliability (Cronbach's alpha), 2) content validity and conceptual arguments, and 3) comments by respondents and missing data. For each subscale item selection was bound to preserve reliability, with Cronbach's alpha above 0.70 [25] and a minimum of four items per subscale.

All analyses were performed in SPSS 17.0 and LISREL 8.80.

## Results

### Sample characteristics

The sample (N = 112) consisted of 45 former project leaders (42%) and 67 team members (58%). In Table 1 the main characteristics of the sample are listed. Most of the sample was female (77% versus 23% males). Most respondents had been employed in the organization for more than 6 years (81%). Half of the respondents (65%) work approximately 30-full time hours per week and 34% works less than 30 hours per week. As to job positions, the largest groups were management staff (44%) and nurses (23%), the smallest were medical assistants (2%) and medical/social specialists (3%). Please note that the category 'Management staff' included team leaders as well as other management positions. Further information on the improvement teams in the improvement program Care for Better can be found elsewhere [18,26,27]. The researcher's communication with the contact persons, who were mostly former project leaders, may have caused the predominance of managers. All improvement projects were represented in the sample. The majority were in the client autonomy project (28%). Others were from: eating and drinking (15%), pressure ulcer care (14%), medication safety (13%). Only a few teams were from prevention of sexual abuse (9%).

**Table 1:** Descriptive statistics of the sample

Age		Mean	SD
	Age in years	45.2	9.3
		(min. 19 - max. 62)	
Gender		Freq.	%
	Male	24	23
	Female	80	77

Average workweek		Freq.	%.
	8-15 hours	2	1.7
	16-22 hours	10	8.4
	23-29 hours	29	24.4
	30-36 hours	63	52.9
	37 hours or more	15	12.6

Number of years in the organization		Freq.	%
	<2 years	1	0.8
	2-3 years	8	6.8
	4-5 years	13	11.0
	6-10 years	35	29.7
	10< years	61	51.7

Position		Freq.	%
	medical assistants	2	1.7
	Nurses	27	23.1
	social workers	14	12.0
	medical/social specialists	3	2.6
	Management	52	44.4
	health policy and quality staff	13	11.1
	para-/perimedical professionals	6	5.1
	clients and representatives	0	0

Role in improvement team		Freq.	%
	project leader	45	41.7
	team member	67	58.3

Number of respondents per program domain		Freq.	%
	Pressure Ulcer Care	18	14.2
	Eating and Drinking	19	15.0
	Prevention Sexual Abuse	11	8.7
	Client Autonomy	35	27.6
	Medication Safety	17	13.4
	Fall Prevention	14	11.0
	Prevention of (Social) Behavioral Problems	13	10.2

### Data preparation and screening

All 52 items were included in the initial modeling phase of the analysis. For each item descriptive statistics were calculated to screen univariate and bivariate normality, and to detect outliers. Some items had more than 20% missing values- we will reflect on this in the discussion. Skewness and/or kurtosis were seen for some items, but no extreme values were found.

#### Modeling phase 1: the initial version & selection for the long version

We start this section with the results of the measurement model for the items and the subscales. Table 2 reports the descriptive statistics for each item and the factor loadings of the initial modeling. Table 3 reports the goodness of fit indices for each version of the instrument. Table 4 reports the descriptive statistics and reliability coefficients for each subscale for each version of the instrument.

**Table 2:** Descriptive statistics per item and factor loadings initial model1°

**No**.	Scale	N	M	SD	λ^§^^
	**Routinization I**				
1*^#^	The new practice is regarded as the standard way to work.	100	3.5	0.9	0.74
2*^#^	The new work practice is easy to describe.	102	3.8	0.7	0.46
3	We have developed variations on the new work practice for different situations.	96	3.2	1.0	0.29
4	The new work practice is hard to pass on to others.	100	3.8	0.7	0.20
5*^#^	All colleagues involved in the new work practice are knowledgeable about it.	99	3.4	0.9	0.73
6*	Everybody has developed their own way to perform the new work practice properly.	100	3.3	0.9	0.57
7*^#^	The work practice has replaced the old routine once and for all.	99	3.3	1.0	0.76
8*	Everyone knows exactly for which tasks and responsibilities they are accountable.	98	3.7	0.7	0.58
9*	Despite the usual exceptions in practice it is not hard to perform the work practice as prescribed.	96	3.3	0.8	0.43
10*^#^	Performing the new routine always goes swimmingly well.	96	2.8	0.8	0.57

	**Routinization II**				
11*^#^	There is little opportunity to adapt the work practice to specific situations.	97	3.6	0.8	0.47
12	The performance is robust even considering external influences outside our control.	91	2.9	0.8	-0.17
13*^#^	We are accustomed to the work practice.	94	3.5	0.9	0.85
14	By performing it the work method continuously changes.	99	3.0	0.9	0.02
15	The exact manner of performing the work practice differs per care team.	94	3.2	1.0	-0.13
16*^#^	We automatically work according to the new work practice.	96	3.3	0.9	0.71
17	Depending on the situation we adapt the way we perform the work practice.	94	3.5	0.8	0.34
18*^#^	We have adjusted our old habits to the new work practice.	96	3.4	0.9	0.54

	**Routinization III: feedback**				
19*^#^	If my work is not up to standard, my colleagues will comment on this.	95	3.4	0.8	0.47
20*^#^	We all keep an eye on potential flaws in the performance.	96	3.8	0.6	0.50
21*^#^	Problems in performing the work practice are usually brought up by our team leader.	94	3.4	0.8	0.58
22	Practical ideas for improving the work practice are rarely exchanged among colleagues.	95	3.4	0.9	0.24
23*^#^	We often jointly discuss how to handle comments.	90	3.4	0.8	0.48

	**Institutionalization of Skills**				
24*	Work practice knowledge and skills are listed in the job requirements in recruitment ads.	88	3.1	1.0	0.56
25*^#^	Newly recruited staff is thoroughly introduced to the work practice.	95	3.4	0.9	0.74
26	Our organization expects that all staff can perform the work practice.	98	3.6	0.8	0.32
27*^#^	We regularly train all staff in the required skills.	102	3.2	0.9	0.73
28*	Occasionally we set up activities to refresh important skills and knowledge.	97	3.1	1.0	0.59
29*^#^	Important knowledge and skills are addressed in performance interviews.	87	3.1	0.9	0.83
30*^#^	Knowledge and skills for the work practice are listed in our job descriptions	88	3.1	1.0	0.74
31*^#^	In performance interviews goals are set for work practice skill development.	88	3.0	0.9	0.79

	**Institutionalization of Documentation Materials**				
32*	All staff is informed that work practice documentation is available.	97	2.9	1.0	0.49
33*	Documentation is accessible to everybody.	100	3.9	0.7	0.40
34*^#^	Work practice documentation is always kept in a special place.	99	3.8	0.8	0.59
35*^#^	Documentation is easily replaced when lost.	89	3.6	0.9	0.64
36	Documentation is always distributed to new colleagues.	82	2.9	0.9	0.36
37	Documentation is not always kept up to date.	93	3.5	0.7	0.18
38*^#^	Documentation is used frequently.	96	3.5	0.8	0.72
39*^#^	Work practice documentation is regularly updated following new developments in (long-term) care.	96	3.6	0.8	0.69
40*^#^	Documentation is used for updating training.	91	3.6	0.9	0.76

	**Institutionalization of Practical Materials**				
41*^#^	Materials are almost always available.	96	4.0	0.7	0.45
42*^#^	Materials are never in the same place.	92	3.8	0.8	0.61
43*^#^	Materials are well-stocked when needed.	91	3.8	0.7	0.67
44	Our materials are often defective.	90	3.9	0.6	0.24
45	Usually materials are replaced when damaged or lost.	86	3.7	0.7	0.27
46*	We always order materials too late.	85	3.7	0.7	0.43
47*^#^	Responsibility for the materials is assigned to designated staff.	90	3.7	0.8	0.61

	**Institutionalization of Team Reflection**				
48*^#^	The new work practice is a regular topic in team meetings.	98	2.9	1.0	0.68
49*^#^	In our team meetings we choose our improvement goals together.	95	3.3	0.9	0.74
50*^#^	The performance of the work practice is evaluated every now and then (for example once per 3 or 6 months).	96	3.3	1.0	0.83
51*^#^	In our team meetings we analyze if we have achieved our improvement goals.	97	3.3	0.9	0.81
52*	Team decisions about the work practice are recorded and made available in minutes or otherwise.	96	3.7	0.8	0.57

° For the hierarchical two factor model. ^§ ^λ = the estimated factor loading for the item. ^ Results for the structural equations per item are available upon request. * Items selected for the long version. ^# ^Items selected for the short version.

N.B. The long and the short version are also listed in Additional file 1.

**Table 3:** Goodness-of-fit indices for the hierarchical CFA

	Model°	likelihood ratio χ^2^*	df	RMSEA (90% C.I.)	CFI	SRMR
**INITIAL MODEL: 52 variables**	0F	2382	1253	0.085 (0.079; 0.090)	0.90	0.10
	1F	2459	1267	0.086 (0.081; 0.092)	0.89	0.11
	2F	2436	1266	0.086 (0.080; 0.091)	0.90	0.10

**Model phase 1:** **LONG selection**	0F	1225	719	0.075 (0.068; 0.082)	0.94	0.08
	1F	1297	733	0.078 (0.071; 0.085)	0.93	0.10
	2F	1262	732	0.076 (0.069; 0.083)	0.94	0.09
**Rerun with non-imputed data**	2F	1059	732	0.096 (0.083; 0.11)	0.87	0.12

**Model phase 2:** **SHORT selection**	0F	642	384	0.073 (0.063; 0.083)	0.95	0.07
	1F	717	398	0.080 (0.070; 0.089)	0.95	0.10
	2F	681	397	0.075 (0.066; 0.085)	0.95	0.08
**Rerun with non-imputed data**	2F	537	397	0.084 (0.065; 0.10)	0.93	0.11

* For all likelihood ratio χ^2^: p < .00001 ° See methods section for the description of the model structures. 0F = basic model with seven factors; 1F = seven factors and one hierarchical latent factor; 2F = proposed structure of seven factors and two hierarchical latent factors, see also Figure 1.

**Table 4:** Descriptive statistics of subscales^a^

	Rout I	Rout II	Rout III	Skills	Docu	Mat	Refl
***Initial model*** **(52 items)**							
**# items**	10	8	5	8	9	7	5
**N**	85(24%)	81(28%)	88(21%)	69(38%)	71(37%)	80(29%)	91(19%)
**Item mean¤**	3.38	3.31	3.50	3.26	3.51	3.8	3.3
**Item variance**	0.73	0.71	0.63	0.83	0.74	0.49	0.82
**Scale mean**	33.8	26.4	17.5	26.1	31.6	26.5	16.6
**Scale SD**	5.4	3.4	2.6	5.7	5.2	3.3	3.7
**Theoretical range**	0 - 50	0 - 40	0 - 25	0 - 40	0 - 45	0 - 35	0 -25
**Average inter-item correlation** **(min.; max.)**	0.34 (-09;.71)	0.15 (-21;.61)	0.29 (-01;.48)	0.56 (.18; 89)	0.39 (-.22;.75)	0.37 (.09; .66)	0.58 (.38; .73)
**Cronbach's alpha**	0.83	0.58	0.67	0.91	0.85	0.80	0.87

***Long version*** **(40 items)(**							
**N**	89	91	88	70	81	81	91
**Score range**	8-36	4-18	4-17	7-33	7-34	6-25	5-21
**Mean**	27.1	13.7	14	22.4	25.1	19	16.6
**SD**	4.9	2.5	2.3	5.6	4.5	2.8	3.7
**# items**	8	4	4	7	7	5	5
**Items included**	1,2,5-10	13,16, 18,11	19-21, 23	24,25, 27-31	32-35, 38-40	41-43, 46,47	48-52
**Cronbach's alpha**	0.86	0.70	0.71	0.93	0.87	0.82	0.87

**Short version** **(30 items)**							
**N**	90	91	88	74	83	86	92
**Score range**	5-23	3-14	4-17	5-23	5-25	4-20	4-17
**Mean**	16.9	10.2	14	16.2	18.2	15.4	12.9
**SD**	3.5	2.1	2.3	4.1	3.5	2.3	3.2
**# items**	5	3	4	5	5	4	4
**Items included**	1,2,5,7, 10	13,16, 18 (11)	19-21, 23	25,27-31	34,35, 38-40	41-43, 47	48-51
**Cronbach's alpha**	0.85	0.75	0.71	0.92	0.89	0.81	0.87
**Correlation with long version^1^**	0.95	0.96	0.93	0.98	0.96	0.98	0.98
	N = 115	N = 111	N = 107	N = 105	N = 105	N = 104	N = 111

^a^rout I = Routinization I. rout II = Routinization II. rout III = Routinization III. Skills = Institutionalization of Skills. Docu = Institutionalization of Documentation Materials. Mat = Institutionalization of Practical Materials. Refl = Institutionalization of Team Reflection. ¤ is the average mean and average variance on the items of a given subscale. ^1 ^for all r, p < .01

The first model tested was a confirmatory hierarchical two factor model with 52 items on the imputed data. On the whole, the factor loadings of the individual items exceeded commonly recommended critical values [20,25,28]. The average factor loadings of the items were high, (average λ = 0.54); for Routinization I, Institutionalization of Skills, Documentation Materials and Team Reflection subscales higher than 0.50, with the exception of Routinization III (average λ = 0.46), Institutionalization of Practical Materials (average λ = 0.47), and Routinization II (average λ = 0.33). Also, the structure coefficients were high (mean = 0.84, range: 0.68 - 1.0) indicating strong relatedness of the variables to the first order factors and thus indicating strong construct validity.

As shown in Table 3, the RMSEA values are just below the critical value of 0.08; the CFI and the SRMR are also low with values around 0.90 and 0.10 for the SRMR. These results suggest that the fit of the initial three models needs improvement, both in relation to variance in the population as well as in relation to the independence model. Comparing the hierarchical one factor model with the hierarchical two factor model, the latter yielded better goodness-of-fit in terms of the -2 Log Likelihood ratio χ^2 ^and the SRMR. For the RMSEA and the CFI no difference was seen between the one factor and the two factor model. As can be expected, a comparable pattern of factor loadings was found in all three models. In Table 2, we reported the factor loading for the hierarchical two factor model because of its better goodness-of-fit.

Next, the internal consistencies of the subscales were computed; see Table 4. All subscales had satisfactory internal consistency.

### Item selection for the long version

Seeing the results of the initial modeling and according to our theoretical model, we decided to base item selection on the estimations for the confirmatory hierarchical two factor model with seven first order factors. For all subscales but Routinization II, we only selected items with a factor loading higher than 0.40.

The following items were included for each subscale: for Routinization I (7 items): 1, 2, and 5 - 10; for Routinization II four items, 11, 13, 16 and 18; for Routinization III four items 19 - 21 and 23; for Institutionalization of Skills seven items 24, 25 and 27 - 31, for Institutionalization of Documentation seven items 32 - 35 and 38 - 40; for Institutionalization of Practical Materials five items 41 - 43 and 46, 47; and for Institutionalization of Team Reflection all five items were selected. By this method, all subscales could be created straightforward -- with the exception of Routinization II.

The items of Routinization II related to each other in various, often inconsistent ways. This is why several explorative analyses were performed with items for other sub dimensions, in particular Routinization I and III. We selected four items with positive factor loadings higher than 0.30. Item 11 did not have the best psychometric properties. However for conceptual reasons it is important and therefore we recommend it should be included. The selected items are indicated with an asterisk in Table 2.

Next, to further assess validity in the form of the structure of the underlying constructs, the SEM analyses were repeated with the long version (see Table 3). As expected, the two factor model yielded better goodness of fit in terms of the -2 Log Likelihood ratio χ^2^, RMSEA, CFI and SRMR compared to the hierarchical one factor model. Also, the values for the fit indices clearly improved compared to the initial modeling. The -2 Log Likelihood ratio χ^2 ^is significantly reduced. The RMSEA and the SRMR conform to the critical values. The CFI value is positive, indicating good fit compared to the independence model. In sum, the hierarchical two factor model prevailed and the model fit was improved but clearly still leaves room for improvement.

As can be seen in Table 4, reliability coefficients for the subscales with selected items ranged from 0.70 (for Routinization II) to 0.93 (for Institutionalization of Skills). This indicates good to excellent internal consistency.

#### Modeling phase 2: construction of a short version

The descriptive statistics and item selection for the short version are included in Table 4. Basic criterion for inclusion is a factor loading higher than 0.40, other reasons for selection are stated when relevant (see also methods section for the criteria).

For the routinization subscales the following selections resulted: for Routinization I five items: 1, 2, 5, 7, and 10; for Routinization II three items: 13, 16 and 18; for Routinization III unchanged selection: 19 - 21, and 23 (since the internal consistency drops to .64 if we removed item 20). For the institutionalization subscales the following selections resulted: for Institutionalization of Skills five items: 25, 27, 29 - 31 (no item needed to be excluded- only item 24 and 28 have somewhat lower factor loadings and were therefore found dismissible); for Institutionalization of Documentation five items: 34, 35, 38, 39 and 40; for Institutionalization of Practical Materials four items: 41 - 43 and 47 (no item needed to be excluded, only item 46 appeared to cross load and therefore was excluded); for Institutionalization of Team Reflection four items: 48, 49, 50 and 51. The selected items are indicated with a hash in Table 2. In Additional file 1 the items of the long and the short version are listed.

The analysis of the hierarchical two factor model repeated with the short version. As can be seen in Table 3, all fit indices improved compared to the long version. We note that for the one factor model the model fit did not improve, as the RMSEA increased and the SRMR remained stable. In consequence, the hierarchical two factor model again performed better than the hierarchical one factor model. Seeing the CFI value for the hierarchical two factor model, i.e. comparing with the independence model, the model fit now is adequate. The RMSEA and SRMR are better than the long version, but also indicate only moderate model fit.

To verify the results with the imputation of data the three models were also computed with the proposed selection of items with non-imputed data for both the long and the short version. These results were similar to the estimations based on imputed data, see Table 3.

Finally, the bivariate correlations between the subscales, based on the short version, were analyzed; the results are fully listed in Additional file 4. Analysis revealed that the subscales correlate significantly (all p-values < .05). Within the routinization dimension r ranged between 0.60 and 0.80. Within the institutionalization dimension r ranged between 0.49 and 0.70, with the exception of the correlation between Institutionalization of Practical Materials and Team Reflection, r = 0.30. The subscales also correlated moderate - high between the two dimensions, r-coefficients ranged between 0.29 and 0.74. The bivariate correlation between total scores for Routinization (three subscales summated) and Institutionalization (four subscales summated) was also strong, r = 0.79. Last, the bivariate correlations were computed between the short version and the long version, the results are included in Table 4. All correlation coefficients are high (range 0.93 - 0.98).

## Discussion

In this paper, we presented a framework and a measurement instrument for the sustainability of changed work practices. The measurement instrument was developed and tested in a follow up study of a quality collaborative program for long-term care. The results will now be discussed in three sections. In the first section, we reflect on the measurement modeling and the construction of the long and short version. The second part addresses the analyses of dimensionality and the theoretical implications of our study. Finally we take into consideration some methodological issues with regard to future use of the framework and the instrument.

### Measurement models

The construct validity of the subscales was supported by the overall positive and high estimates for both item factor loadings and structure coefficients. In addition, the reliability coefficients of the subscales well exceeded the criterion of 0.70. In other words, the evidence supports both the validity and reliability of the instrument. As a result, we were able to construe a long and a short version with good psychometric properties. Given the strong correlations between the long and the short version of each subscale we recommend using the short version. In case one is interested in one or more specific sub dimensions, the long version is more appropriate.

The measurement model revealed some difficulties for the sub scale Routinization II. Several items cross loaded and for some items the factor loadings were very low. Routinization II centers on variations in practice and if the practices have led to new variations in the principles. It is possible that for some items, the interpretation of the items was problematic. For example, think of variation in practices -- is it a good sign or a bad sign in terms of sustainability? For some respondents, a positive answer may have seemed risky given their professional norms. This may have been especially the case for respondents with managerial functions, who were overrepresented in our sample.

In the three subscales for routinization, we differentiated several aspects of the dynamic, bidirectional relations between principles and practices described by Feldman and Pentland [3]. Support for the distinctions between these sub dimensions is found in the bivariate correlations where we saw positive relationships but also some pronounced differences, especially in the relationships with the sub dimensions for Institutionalization. This can be taken as an indication of the importance of different forms of organizational learning for routinization, enabled by different aspects of the institutions created for the work practice [8-10].

### Sustainability and the analysis of the two dimensions

For lack of a theoretical conceptualization, we extended the work of Feldman and Pentland [3] on organizational routines to the domain of quality improvement in health care. We have conceived sustainability as a dynamic process in which organizational routines are cultivated through routinization and institutionalization. These concepts were further elaborated in relation to Yin's work on sustainability [6,7]. Dimensionality was tested by comparing a hierarchical two factor model with a hierarchical one factor model. The two factor model yielded the best model fit. At the same time the subscales were found to relate positively to each other. These findings illustrate the internal validity of the instrument and substantiate that the dimensions - and their sub dimensions- reflect different yet related aspects of sustainability. They also underline the value of multidimensional constructs in this domain: the nature and influence of the dimensions may vary between work practices, quality problems, interventions, and organizational contexts. Second, these results show the usefulness of evaluating (changed) work practices in terms of organizational routines- an approach not often applied in health care. As most scholars approach sustainability as rather static, we hope the application of routine theory to this domain is beneficial not only in explaining everyday variations in practice, but also certain implementation problems, evaporation and decay of innovations [1,29-31].

The results illustrate that institutional theory has much to offer to the study of quality improvement in health care. Although the concept of institutionalization is not new to the study of sustainability of work practices, the strength of our work lies in the way we have operationalized it. In the four dimensions, we can recognize aspects of institutions, making the process of institutionalization tangible. We realize that institutional theory is deployed in many scholarly contexts to describe a multitude of processes, structures and practices, influencing each other at different levels (macro-, meso-, and microlevels) [32,33]. Our approach is centred on the micro level of a work practice and on what it takes to organize it locally- thus within health care organizations or even within their departments. Noting this is relevant to contextualize how we use the concept. Moreover, in our approach both concepts are dynamic. Thus, although the processes of institutionalization may yield temporarily stable structures and processes, we do not regard these as inherently static. However, within institutional theory, there are debates on the extent to which institutionalization may entail rigidity of structures and processes -- as opposed to flexibility and change.

Last, the framework with its sub dimensions may not only be applicable to long-term care, but also to hospital care or even to service organizations outside health care. It could serve to make visible some of the results of quality improvements that now remain outside the scope of the often used performance or outcome indicators. This may be extra valuable because quality improvement is costly and evaluation has become more and more important given the scarcity of resources available for improvement of services.

### Limitations

We now reflect on some methodological issues with regard to our study.

First, the response rates, and consequently the sample size, were small. As mentioned before, many team members now held other jobs or had left the organization. Furthermore the context of the care organizations participating in the program was very dynamic- many organizations were introducing new (compulsory) policies, reorganizing or even merging. In light of these processes attrition is expected and the resulting response can be considered adequate.

A second limitation regards the use of imputed data. While the EM-algorithm has excellent statistical properties compared to other methods of imputation [34,35] and a rerun for the long and for the short version with non-imputed data yielded highly similar results, still replication with 'complete' data is advised to verify and strengthen the evidence base.

Third, we note that the choice for improvement teams has some disadvantages; for example, it could entail certain biases in the instrument as well as in the evaluation research. Our motive for testing with improvement team members was that they are acquainted with the work practice both before and after intervening. A next step would be to include practitioners who have not taken part in the improvement project. In relation to this, we realize that improvement teams are generally rather highly educated. It is likely that application of the measurement instrument in other professional groups, with lower vocational education, may require some adjustment of the wording of the items.

Fourth, in our study, we have analysed the data on the individual level, which is a common approach to assess validity of measurement instruments. But, we are aware that, in general the perceptions of employees on work practices are interrelated within organizations. Future research should address questions of validity of the instrument on the team or ward level.

Fifth, we mention that although the values we found for internal consistency were sufficient- still it would be better to also assess test-retest reliability.

Last, we reflect on the model fit. The modeling of the long and short version revealed improvement in the model fit but some problems remained, predominantly on the level of residuals (SRMR). This may be due to the choice to restrict cross loading of items.

## Conclusions

In this study we presented a framework and a measurement instrument to assess sustainability of changed work practices after implementation of quality improvements. Sustainability is conceptualized with two dimensions routinization and institutionalization. These dimensions are intimately related, yet they each have distinct value in the discussion of sustainability. Distinguishing between routinization and institutionalization may be fruitful also in relation to other forms of sustainability, such as results, improvement practices/capacity, as well as aspects of improvement processes. The psychometric properties of the measurement instrument warrant application of the instrument in the evaluation of improvement projects.

## Competing interests

The authors declare that they have no competing interests.

## Authors' contributions

SS participated in the design, developed the framework and the measurement instrument, participated in the data collection, performed the statistical analyses and drafted the manuscript. MS conceived of the study, participated in the design and the data collection, assisted the statistical analyses and helped to draft the manuscript. RB conceived of the study, participated in the design and reviewed the manuscript. AN conceived of the study, participated in the design and its coordination, advised on the statistical analysis, and helped to draft the manuscript. The co-authors have approved the submitted version of the manuscript and accept responsibility for the data presented.

## Pre-publication history

The pre-publication history for this paper can be accessed here:

http://www.biomedcentral.com/1472-6963/11/314/prepub

## Supplementary Material

Additional file 1 **Measurement instrument for sustainability: initial, long and short version**. the file contains a list of the items for the initial, long and short version of the measurement instrument for sustainability of work practices. Click here for file

Additional file 2 **PCA results**. the file contains the results of principal component analyses for the two dimensions Routinization and Institutionalization. Click here for file

Additional file 3 **NNFI/Tucker-Lewis indices for the hierarchical CFA in SEM**. the file contains the SEM results for the NNFI/Tucker-Lewis fit index. Click here for file

Additional file 4 **Correlations between subscales - based on the short version**. the file contains the results of bivariate correlation analyses for the seven subscales for sustainability. Click here for file
