# Supplementary material for: A framework and a measurement instrument for sustainability of work practices in long-term care
Source: BMC Health Serv Res. 2011 Nov 16;11:314. doi: 10.1186/1472-6963-11-314 (PMC3234291; doi:10.1186/1472-6963-11-314)
Supplement: Additional file 1 — Measurement instrument for sustainability: initial, long and short version. the file contains a list of the items for the initial, long and short version of the measurement instrument for sustainability of work practices. [file 1472-6963-11-314-S1.PDF]

Additional file 1. Initial, long and short version of the measurement instrument for sustainability of changed work practices

	**INITIAL version**
	**Routinization I**
1.	The new practice is regarded as the standard way to work.
2.	The new work practice is easy to describe.
3.	We have developed variations on the new work practice for different situations.
4.	The new work practice is hard to pass on to others.
5.	All colleagues involved in the new work practice are knowledgeable about it.
6.	Everybody has developed their own way to perform the new work practice properly.
7.	The work practice has replaced the old routine once and for all.
8.	Everyone knows exactly for which tasks and responsibilities they are accountable.
9.	Despite the usual exceptions in practice, it is not hard to perform the work practice as prescribed.
10.	Performing the new routine always goes swimmingly well.
	**Routinization II**
11.	There is little opportunity to adapt the work practice to specific situations.
12.	The performance is robust even considering external influences outside our control.
13.	We are accustomed to the work practice.
14.	By performing it the work method continuously changes.
15.	The exact manner of performing the work practice differs per care team.
16.	We automatically work according to the new work practice.
17.	Depending on the situation we adapt the way we perform the work practice.
18.	We have adjusted our old habits to the new work practice.
	**Routinization III: feedback**
19.	If my work is not up to standard, my colleagues will comment on this.
20.	We all keep an eye on potential flaws in the performance.
21.	Problems in performing the work practice are usually brought up by our team leader.
22.	Practical ideas for improving the work practice are rarely exchanged among colleagues.
23.	We often jointly discuss how to handle comments.
	**Institutionalization of Skills**
24.	Work practice knowledge and skills are listed in the job requirements in recruitment ads.
25.	Newly recruited staff is thoroughly introduced to the work practice.
26.	Our organization expects that all staff can perform the work practice.
27.	We regularly train all staff in the required skills.
28.	Occasionally we set up activities to refresh important skills and knowledge.
29.	Important knowledge and skills are addressed in performance interviews.
30.	Knowledge and skills for the work practice are listed in our job descriptions
31.	In performance interviews goals are set for work practice skill development.
	**Institutionalization of Documentation Materials***
32.	All staff is informed that work practice documentation is available.
33.	Documentation is accessible to everybody.
34.	Work practice documentation is always kept in a special place.
35.	Documentation is easily replaced when lost.
36.	Documentation is always distributed to new colleagues.
37.	Documentation is not always kept up to date.
38.	Documentation is used frequently.

39. Work practice documentation is regularly updated following new developments in (long-term) care.
40. Documentation is used for updating training.

Institutionalization of Practical Materials*

41. Materials are almost always available.
42. Materials are never in the same place.
43. Materials are well-stocked when needed.
44. Our materials are often defective.
45. Usually materials are replaced when damaged or lost.
46. We always order materials too late.
47. Responsibility for the materials is assigned to designated staff.

Institutionalization of Team Reflection

48. The new work practice is a regular topic in team meetings.
49. In our team meetings we choose our improvement goals together.
50. The performance of the work practice is evaluated every now and then (for example once per 3 or 6 months).
51. In our team meetings we analyze if we have achieved our improvement goals.
52. Team decisions about the work practice are recorded and made available in minutes or otherwise.

* please note: these scales contained special introduction texts and priming question to aid the respondent in understanding the question and relating it to their own work practice. These are included at the end of this Additional file.

	**LONG version**
**No.**	**Routinization I**
1.	The new practice is regarded as the standard way to work.
2.	The new work practice is easy to describe.
5.	All colleagues involved in the new work practice are knowledgeable about it.
6.	Everybody has developed their own way to perform the new work practice properly.
7.	The work practice has replaced the old routine once and for all.
8.	Everyone knows exactly for which tasks and responsibilities they are accountable.
9.	Despite the usual exceptions in practice, it is not hard to perform the work practice as prescribed.
10.	Performing the new routine always goes swimmingly well.
	**Routinization II**
13.	We are accustomed to the work practice.
16.	We automatically work according to the new work practice.
18.	We have adjusted our old habits to the new work practice.
11.	Optional: There is little opportunity to adapt the work practice to specific situations.
	**Routinization III - feedback**
19.	If my work is not up to standard, my colleagues will comment on this.
20.	We all keep an eye on potential flaws in the performance.
21.	Problems in performing the work practice are usually brought up by our team leader.
23.	We often jointly discuss how to handle comments.
	**Institutionalization of Skills**
24.	Work practice knowledge and skills are listed in the job requirements in recruitment ads.
25.	Newly recruited staff is thoroughly introduced to the work practice.
27.	We regularly train all staff in the required skills.
29.	Important knowledge and skills are addressed in performance interviews
30.	Knowledge and skills for the work practice are listed in our job descriptions
31.	In performance interviews goals are set for work practice skill development.
	**Institutionalization of Documentation Materials***
32.	All staff is informed that work practice documentation is available.
33.	Documentation is accessible to everybody.
34.	Work practice documentation is always kept in a special place.
35.	Documentation is easily replaced when lost.
38.	Documentation is used frequently.
39.	Work practice documentation is regularly updated following new developments in (long-term) care.
40.	Documentation is used for updating training.
	**Institutionalization of Practical Materials***
41.	Materials are almost always available.
42.	Materials are never in the same place.
43.	Materials are well-stocked when needed.
46.	We always order our materials too late.
47.	Responsibility for the materials is assigned to designated staff.
	**Institutionalization of Team Reflection**
48.	The new work practice is a regular topic in team meetings.
49.	In our team meetings we choose our improvement goals together.
50.	The performance of the work practice is evaluated every now and then (for example once per 3 or 6 months).
51.	In our team meetings we analyze if we have achieved our improvement goals.
52.	Team decisions about the worm practice are recorded, and made available in minutes or otherwise.

	**SHORT version**
**No.**	**Routinization I**
1.	The new practice is regarded as the standard way to work.
2.	The new work practice is easy to describe.
5.	All colleagues involved in the new work practice are knowledgeable about it.
7.	The work practice has replaced the old routine once and for all.
10.	Performing the new routine always goes swimmingly well.
	**Routinization II**
13.	We are accustomed to the work practice.
16.	We automatically work according to the new work practice.
18.	We have adjusted our old habits to the new work practice.
11.	Optional: There is little opportunity to adapt the work practice to specific situations.
	**Routinization III - feedback**
19.	If my work is not up to standard, my colleagues will comment on this.
20.	We all keep an eye on potential flaws in the performance.
21.	Problems in performing the work practice are usually brought up by our team leader.
23.	We often jointly discuss how to handle comments.
	**Institutionalization of Skills**
25.	Newly recruited staff is thoroughly introduced to the work practice.
27.	We regularly train all staff in the required skills.
29.	Important knowledge and skills are addressed in performance interviews
30.	Knowledge and skills for the work practice are listed in our job descriptions
31.	In performance interviews goals are set for work practice skill development.
	**Institutionalization of Documentation Materials***
34.	Work practice documentation is always kept in a special place.
35.	Documentation is easily replaced when lost.
38.	Documentation is used frequently.
39.	Work practice documentation is regularly updated following new developments in (long-term) care.
40.	Documentation is used for updating training.
	**Institutionalization of Practical Materials***
41.	Materials are almost always available.
42.	Materials are never in the same place.
43.	Materials are well-stocked when needed.
47.	Responsibility for the materials is assigned to designated staff.
	**Institutionalization of Team Reflection**
48.	The new work practice is a regular topic in team meetings.
49.	In our team meetings we choose our improvement goals together.
50.	The performance of the work practice is evaluated every now and then (for example once per 3 or 6 months).
51.	In our team meetings we analyze if we have achieved our improvement goals.

.

Introduction for documentation:

The next scale centres on the use of documentation materials for the changed work practice. Depending on the type of care process, some forms of documentation materials or manuals will be important. There are many kinds of documentation materials in health care. For this reason, we define documentation materials as all (written) resources used for reference or instruction for the changed work practice, such as: protocols, information brochures, books, instructions, user manuals for instruments, and so on.

In connection to the changed work practice, we have:	
Protocols or other guidelines for the care related aspects of the work practice.	☐ yes<br>☐ no
Documentation or resources about organization practices, such as: registration procedures, administration, etc.	☐ yes<br>☐ no
Documentation for instruments or diagnostic tests, etc.	☐ yes<br>☐ no

How important are these for the actual performance?	☐ very important<br>☐ moderately important<br>☐ neutral<br>☐ slightly important<br>☐ not important at all<br>☐ I don't know

Introduction for practical materials:

In long-term care many materials are used daily. There are many different kinds of practical materials, such as: a food car, a blood pressure cuff, incontinence materials, supportive stockings, but also, actual organizational charts or schedules, a list of nutrition prescriptions, and so on. In the following items we are interested in the materials for your changed work practice.

Please take a moment to briefly describe which materials are used in the work practice:
……………………………………………………………………………………………………..<br>……………………………………………………………………………………………………..<br>……………………………………………………………………………………………………..<br>……………………………………………………………………………………………………..

To what extent are the following materials used, when you work according to the changed work practice?	
Medical instruments or tools	☐ very important<br>☐ moderately important<br>☐ neutral<br>☐ slightly important<br>☐ not important at all<br>☐ I don't know
Diagnostic tests	☐ very important<br>☐ moderately important<br>☐ neutral<br>☐ slightly important<br>☐ not important at all<br>☐ I don't know
Organizational instruments	☐ very important<br>☐ moderately important<br>☐ neutral<br>☐ slightly important<br>☐ not important at all<br>☐ I don't know
Care plans or other client related charts	☐ very important<br>☐ moderately important<br>☐ neutral<br>☐ slightly important<br>☐ not important at all<br>☐ I don't know

Other materials are used, namely:
……………………………………………………………………………………………………..<br>……………………………………………………………………………………………………..<br>……………………………………………………………………………………………………..<br>……………………………………………………………………………………………………..
