# Supplementary material for: A framework and a measurement instrument for sustainability of work practices in long-term care
Source: BMC Health Serv Res. 2011 Nov 16;11:314. doi: 10.1186/1472-6963-11-314 (PMC3234291; doi:10.1186/1472-6963-11-314)
Supplement: Additional file 2 — PCA results. the file contains the results of principal component analyses for the two dimensions Routinization and Institutionalization. [file 1472-6963-11-314-S2.PDF]

Additional file 2. PCA results

PCA results for Routinization

Com-ponent	Initial Eigenvalue		Rotated Sums of Squared Loadings			Component Transformation Matrix				
	Total	% of Var.	Total	% of Var.	Cum-ulative %	c1	c2	c3	c4	c5
1	7.9	34.5	5.7	24.9	24.9	0.82	0.52	-0.17	0.09	0.17
2	2.8	12.2	3.2	14.1	39	-0.04	0.27	0.87	0.41	0.02
3	1.4	6.1	2.6	11.4	50.4	-0.47	0.8	-0.19	-0.16	-0.29
4	1.2	5.4	1.6	6.8	57.1	0.07	0.07	0.39	-0.87	0.28
5	1.1	4.7	1.3	5.7	62.9	-0.33	0.14	-0.17	0.2	0.9

	Communalities	Rotated Component Matrix*				
		c1	c2	c3	c4	c5
**Rout I-1**	0.73	0.81	0.15	-0.08	0.01	0.22
**Rout I-2**	0.60	0.54	0.17	-0.45	0.08	0.28
**Rout I-3**	0.71	0.33	0.03	0.43	0.61	0.22
**Rout I-4**	0.59	0.04	0.18	-0.73	0.06	0.11
**Rout I-5**	0.64	0.72	0.29	-0.16	0.10	-0.02
**Rout I-6**	0.58	0.56	0.41	0.25	0.15	0.11
**Rout I-7**	0.68	0.79	0.16	-0.18	-0.06	0.00
**Rout I-8**	0.65	0.37	0.59	-0.16	0.02	0.37
**Rout I-9**	0.50	0.35	0.42	0.09	0.33	0.29
**Rout I-10**	0.62	0.78	-0.03	-0.01	0.02	0.11
**Rout II-1**	0.73	0.36	0.09	-0.22	-0.11	0.73
**Rout II-2**	0.45	-0.08	-0.17	0.64	0.02	-0.09
**Rout II-3**	0.81	0.85	0.22	-0.02	0.07	0.17
**Rout II-4**	0.73	-0.06	0.37	0.66	0.28	0.28
**Rout II-5**	0.58	-0.20	0.18	0.71	0.09	-0.03
**Rout II-6**	0.61	0.62	0.42	-0.22	0.03	0.04
**Rout II-7**	0.58	0.26	0.43	0.07	0.57	-0.08
**Rout II-8**	0.54	0.53	0.45	-0.22	0.08	0.06
**Feedback-1**	0.60	0.47	0.44	-0.15	0.12	-0.39
**Feedback-2**	0.67	0.55	0.55	0.06	-0.23	0.00
**Feedback-3**	0.63	0.39	0.66	-0.09	0.00	-0.18
**Feedback-4**	0.63	0.20	0.19	0.05	-0.73	0.15
**Feedback-5**	0.61	0.05	0.77	0.02	-0.03	0.12

- Method: Varimax rotation with Kaiser normalization.

PCA results for Institutionalization

	Initial Eigenvalues		Rotated Sums of Squared Loadings			Rotated Structure Matrix							
**Com-ponent**	**Total**	**% of Var**	**Total**	**% of Var.**	**Cum-ulative %**	**c1**	**c2**	**C3**	**c4**	**c5**	**c6**	**c7**	**c8**
**1**	11.51	39.7	5.50	18.95	18.95	0.62	0.43	0.39	0.42	0.15	0.21	0.15	0.11
**2**	2.69	9.28	3.63	12.51	31.46	-0.34	0.39	-0.54	0.33	0.53	0.06	0.21	-0.09
**3**	2.04	7.05	3.33	11.47	42.94	0.30	-0.57	-0.23	0.11	0.19	-0.23	0.52	0.41
**4**	1.56	5.36	3.21	11.07	54.00	-0.42	-0.07	0.43	0.13	0.37	-0.02	-0.31	0.62
**5**	1.42	4.89	1.92	6.63	60.64	-0.05	-0.41	0.01	0.00	0.14	0.89	0.03	-0.13
**6**	1.35	4.64	1.89	6.53	67.17	-0.34	0.12	0.50	-0.29	0.08	-0.03	0.71	-0.19
**7**	1.25	4.32	1.83	6.32	73.49	-0.04	0.38	-0.28	-0.32	-0.39	0.32	0.20	0.61
**8**	1.13	3.89	1.64	5.65	79.13	0.34	0.11	-0.06	-0.70	0.59	-0.02	-0.17	0.00

		Rotated Component Matrix							
	**Communalities**	**c1**	**c2**	**c3**	**c4**	**c5**	**c6**	**c7**	**c8**
**Skills-1**	0.64	0.52	-0.08	0.37	0.35	-0.07	-0.30	0.04	0.09
**Skills-2**	0.86	0.63	0.12	0.20	0.42	0.00	0.39	0.28	0.04
**Skills-3**	0.83	0.21	-0.23	0.02	0.31	-0.09	0.36	0.59	0.38
**Skills-4**	0.83	0.74	0.16	0.17	0.14	0.15	0.44	0.00	0.03
**Skills-5**	0.73	0.77	0.24	0.15	0.11	-0.09	0.11	-0.14	0.08
**Skills-6**	0.86	0.77	0.10	0.21	0.18	0.19	0.07	0.37	-0.04
**Skills-7**	0.80	0.68	0.06	0.48	0.12	0.11	0.20	0.16	-0.07
**Skills-8**	0.86	0.71	0.14	0.33	0.18	0.14	-0.06	0.41	-0.13
**Skills-9**	0.87	0.21	0.21	0.19	0.83	0.11	0.20	0.04	-0.06
**Materials-1**	0.63	0.01	0.31	0.02	0.61	0.33	0.10	0.16	0.14
**Materials-2**	0.81	0.43	0.27	0.03	0.73	0.08	-0.05	0.09	0.01
**Materials-3**	0.77	0.09	0.05	-0.06	0.06	0.86	0.00	0.02	0.11
**Materials-4**	0.71	0.09	0.19	-0.09	0.02	0.14	-0.03	0.80	0.01
**Materials-5**	0.82	0.05	0.19	0.05	0.34	0.80	0.06	0.12	0.03
**Materials-6**	0.71	0.33	0.27	0.24	0.59	0.30	0.13	-0.08	-0.03
**Materials-7**	0.62	0.21	0.22	0.10	0.28	0.09	0.62	0.06	0.21
**Documentation-1**	0.88	0.19	0.79	0.18	0.18	0.05	0.26	-0.13	-0.27
**Documentation-2**	0.86	0.20	0.75	0.10	0.39	0.17	0.24	0.15	-0.02
**Documentation-3**	0.72	0.09	0.70	0.16	0.25	0.26	0.03	0.25	0.06
**Documentation-4**	0.71	0.19	0.47	0.39	0.12	-0.15	-0.35	0.37	0.09
**Documentation-5**	0.87	0.01	-0.01	0.12	-0.05	0.19	0.04	0.12	0.89
**Documentation-6**	0.81	0.50	0.62	0.19	0.23	0.02	0.20	0.12	0.16
**Documentation-7**	0.85	0.58	0.49	0.07	0.32	-0.06	-0.05	-0.10	0.39
**Documentation-8**	0.84	0.69	0.54	0.02	0.11	0.12	0.07	-0.01	0.20
**Reflection-1**	0.76	0.44	0.02	0.46	0.24	-0.09	-0.03	-0.26	0.47
**Reflection-2**	0.80	0.27	0.06	0.85	0.02	0.01	0.08	-0.03	0.03
**Reflection-3**	0.84	0.45	0.12	0.72	0.26	0.02	0.13	0.07	0.10
**Reflection-4**	0.87	0.07	0.32	0.84	0.09	0.00	0.18	-0.07	0.12
**Reflection-5**	0.81	0.20	0.34	0.41	0.02	-0.05	0.68	-0.03	-0.17
