# Supplementary material for: A framework and a measurement instrument for sustainability of work practices in long-term care
Source: BMC Health Serv Res. 2011 Nov 16;11:314. doi: 10.1186/1472-6963-11-314 (PMC3234291; doi:10.1186/1472-6963-11-314)
Supplement: Additional file 3 — NNFI/Tucker-Lewis indices for the hierarchical CFA in SEM. the file contains the SEM results for the NNFI/Tucker-Lewis fit index. [file 1472-6963-11-314-S3.PDF]

Additional file 3. NNFI / Tucker-Lewis Indices for the modeling

	Model°	NNFI
**INITIAL MODEL: 52 variables**	0F	0.89
	1F	0.89
	2F	0.89
**Model phase 1: LONG selection**	0F	0.93
	1F	0.93
	2F	0.93
Non-imputed data	2f	0.86
**Model phase 2: SHORT selection**	0F	0.95
	1F	0.94
	2F	0.95
Non imputed data	2F	0.93

° See methods section for the description of the model structures. 0F= basic model with seven factors; 1F= seven factors and one hierarchical latent factor; 2F= proposed structure of seven factors and two hierarchical latent factors, see also Figure 1.

The NNFI also is an incremental / comparative fit index, however it corrects for model complexity (i.e. it favors simpler models) [20].

Results. The initial modeling shows that based on the NNFI can not distinguish between the three models. In relation to the critical value of .90 it is clear the initial model leaves room for improvement. The modeling with the long version then reveals better a model fit. Again no differences between the three models are seen. Finally, the short version was modeled. This yielded improved model fit, meaning that the differences between the independence model and the estimated model have decreased. Now is also becomes apparent that the one factor model does not perform as well. In conclusion we note that based on these results the question if sustainability is best described with the two dimensional model is partially confirmed.
