# Supplementary material for: A framework and a measurement instrument for sustainability of work practices in long-term care
Source: BMC Health Serv Res. 2011 Nov 16;11:314. doi: 10.1186/1472-6963-11-314 (PMC3234291; doi:10.1186/1472-6963-11-314)
Supplement: Additional file 4 — Correlations between subscales - based on the short version. the file contains the results of bivariate correlation analyses for the seven subscales for sustainability. [file 1472-6963-11-314-S4.PDF]

Additional file 4. Correlations between subscales- based on short version

		Rout I	Rout II	Rout III	Skills	Docu	Mat	Refl
**Rout I**	r		0.84*	0.66*	0.71*	0.68*	0.53*	0.48*
	N		85	80	62	71	72	83
**Rout II**	r			0.59*	0.65*	0.58*	0.57*	0.44*
	N			81	65	70	72	85
**Rout III**	r				0.64*	0.49*	0.49*	0.57*
	N				62	73	72	81
**Skills**	r					0.68*	0.50*	0.70*
	N					60	57	65
**Docu**	r						0.59*	0.52*
	N						67	71
**Mat**	r							0.34*
	N							72
**Refl**	r							
	N							

Note. * p<.01. [a]rout I = Routinization I. rout II = Routinization II. rout III = Routinization III. Skills = Institutionalization of Skills. Docu = Institutionalization of Documentation Materials. Mat = Institutionalization of Practical Materials. Refl = Institutionalization of Team Reflection
